# Intracranial Aneurysm Biomarkers: A Convergence of Genetics, Inflammation, Oxidative Stress, and the Extracellular Matrix

**DOI:** 10.3390/ijms26073316

**Published:** 2025-04-02

**Authors:** Enhao Zhang, Xu Yan, Hangyu Shen, Mingyue Zhao, Xiang Gao, Yi Huang

**Affiliations:** 1 Ningbo Key Laboratory of Nervous System and Brain Function, Department of Neurosurgery, The First Affiliated Hospital of Ningbo University, Ningbo 315010, China; zeh1228@163.com (E.Z.); y15830524126@outlook.com (X.Y.); whale123098@163.com (H.S.); 18360396513@163.com (M.Z.); 2 Key Laboratory of Precision Medicine for Atherosclerotic Diseases of Zhejiang Province, Ningbo 315010, China

**Keywords:** intracranial aneurysm, biomarker, genetics, inflammation, oxidative stress, extracellular matrix

## Abstract

Intracranial aneurysm (IA) is a common cerebrovascular disease in which sacral aneurysms occurring in the Wills ring region can lead to devastating subarachnoid hemorrhage. Despite advances in research, the underlying mechanisms of IA formation and rupture remain incompletely understood, hindering early diagnosis and effective treatment. This review comprehensively summarizes the current landscape of IA biomarkers, encompassing genetic markers, DNA, RNA, inflammatory molecules, oxidative stress proteins, and extracellular matrix (ECM) components. Accumulating evidence suggests that various biomarkers are associated with different stages of IA pathogenesis, including initiation, progression, and rupture. Aberrant ECM composition and remodeling have been observed in IA patients, and extracellular matrix-degrading enzymes are implicated in IA growth and rupture. Biomarker research in IA holds great potential for improving clinical outcomes. Future studies should focus on validating the existing biomarkers, identifying novel ones, and investigating their underlying mechanisms to facilitate the development of personalized preventive and therapeutic strategies for IA.

## 1. Background

**Figure 1:**
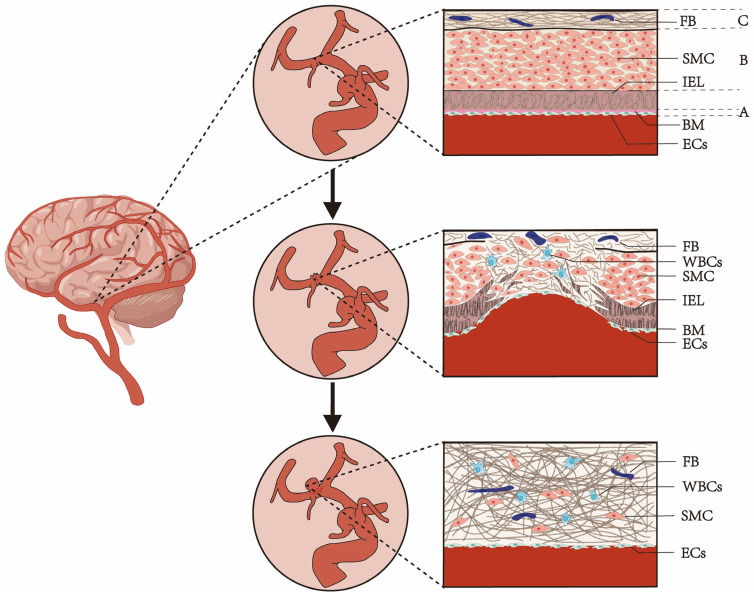
Structural changes during the formation of intracranial aneurysms. Under physiological conditions, the structure of cerebral arteries can be divided into three layers (from the inside out): (**A**) The intima, including the basement membrane (BM) and endothelial cells (ECs). (**B**) The middle membrane is composed of smooth muscle cells (SMCs) surrounding the direction of vessel, embedded with tightly arranged collagen and elastic fiber networks, ensuring good vascular compliance. (**C**) The outer membrane, mainly composed of collagen, maintains the integrity of the vascular wall structure and contains fibroblasts (FBs) and white blood cells (WBCs) [8,9]. There is an inner elastic layer (IEL) with elastic fibers between the intima and media, which is considered a key structure leading to the formation of aneurysms. The anatomical variations, structural composition, and physiological homeostasis of the cerebral arterial wall may be influenced by risk factors for intracranial aneurysm development, all of which contribute to abnormal blood flow. In response to these risk factors, structural changes in the cerebral arterial wall result in the disruption of the IEL at arterial bifurcations. Abnormal blood flow causes mechanical overload and tension transfer, leading to the continuous reconstruction and degradation of the extracellular matrix through SMC apoptosis or regulation as well as endothelial cell dysfunction and macrophage influx [10,11]. Blood inflow and impingement expose structurally deficient arteries to high wall shear stress, leading to the formation of an aneurysmal sac. The aneurysmal sac continues to grow until a balance is reached between continuous vascular wall repair and extracellular matrix degradation [12]. The main cellular components in the aneurysm wall are SMCs, a discontinuous layer of ECs, and a small number of inflammatory cells such as neutrophils, macrophages, and lymphocytes [9,13].

Intracranial aneurysm (IA), a prevalent cerebrovascular disease, occurs in 1–2% of the population [1]. It mainly occurs in the arterial system at the bottom of the brain, especially within the Willis ring, also known as sacral (Berry) aneurysm. The hallmark feature of IA is localized alteration of the cerebral artery wall structure, including the absence of the internal elastic lamina (IEL) and media rupture (Figure 1) [2]. In a cross-sectional study conducted in China, 7% of adults aged 35 to 75 were found to have aneurysms through extensive screening using magnetic resonance angiography (MRA) [3]. In a prevalence study based on a European population, approximately 1.8% of adult participants were found to have aneurysms through screening MRI [4]. Unruptured intracranial aneurysms are more common in women than in men, with a female-to-male ratio of 3:1 in large population studies [5]. They are also more prevalent in the elderly and are rare in children [6]. Untreated IA can rupture any time, with subarachnoid hemorrhage (SAH) being a frequent consequence. Among SAH patients, approximately 12% die before reaching medical attention, 40% die within one month of onset, and over 1/3 of survivors suffer permanent neurological deficits [7].

Due to the high mortality and disability associated with IA rupture, early identification and treatment are crucial. Early IA is often asymptomatic, necessitating early screening for diagnosis. Angiography is a common tool for early detection and diagnosis. Digital subtraction angiography (DSA) remains the gold standard for IA diagnosis; however, it is invasive and time-consuming. Non-invasive techniques like computed tomography angiography (CTA) are increasingly used. While CTA offers increased accessibility, it may lack sensitivity for smaller aneurysms, and it requires radiation exposure and contrast agents, which can be problematic for patients with renal dysfunction or contrast agent allergies [14]. Magnetic resonance angiography (MRA) is another non-invasive method with diagnostic accuracy similar to CTA, and it avoids the aforementioned issues with contrast agents. However, MRA has limitations in specificity [15]. The absence of readily available non-invasive biomarkers for IA diagnosis underscores the need for discovering novel markers to predict IA presence and rupture risk.

## 2. The Risk Factors and Causes of Aneurysm Formation and Rupture

Intracranial aneurysms (IAs) are characterized by localized changes in the structure of the cerebral artery walls. These changes include the absence of the IEL and media rupture. The etiology of IA formation and rupture is complex and not fully understood, but it likely involves a combination of genetic susceptibility, modifiable vascular risk factors (including hypertension, lipid accumulation, atherosclerosis, and smoking), and hemodynamic stress [8].

Healthy cerebral arteries consist of three distinct layers: the intima (innermost), media, and adventitia (outermost) [16]. The IEL, a crucial structure within the media, contributes to IA formation [16]. Compared with other arteries, intracranial arteries have a thicker IEL with a lower proportion of elastic fibers and smooth muscle cells (SMCs) in the media and a thinner adventitia with sparser connective tissue in the subarachnoid space. These anatomical features may render them more susceptible to aneurysm formation [16].

Genetic susceptibility plays a role in IA, with a large-scale meta-analysis identifying 19 single nucleotide polymorphisms (SNPs) associated with sporadic IA [17]. However, the largest twin study to date did not find significant genetic contributions, suggesting a complex interplay between genes and environment [18]. Modifiable risk factors, such as smoking and hypertension, appear to have a more decisive role. A case–control study reported that smoking and hypertension are strong risk factors for IA [19].

Hemodynamic stress is another important factor. IA commonly occurs at arterial bifurcations, branching points, or locations with abrupt changes in vascular geometry, where excessive hemodynamic stress is exerted on the arterial wall [20]. While some studies suggest high wall shear stress (WSS) and WSS gradients contribute to aneurysm formation [20], others suggest low WSS may promote the growth and rupture of large atherosclerotic aneurysms, while high WSS may drive the development and rupture of small aneurysms [21]. This highlights the complex role of hemodynamic forces in IA pathogenesis.

The common steps in IA formation include endothelial dysfunction/injury, exacerbated inflammatory responses, vascular smooth muscle cell (VSMC) phenotype modulation, extracellular matrix remodeling, followed by subsequent cell death and vascular wall degeneration [8]. Risk factors can lead to the rupture of the IEL, causing abnormal blood flow and mechanical stress on the arterial wall. This, in turn, triggers SMC apoptosis or modulation, endothelial dysfunction, and macrophage influx, further promoting inflammatory responses and extracellular matrix degradation [8]. Macrophages play a crucial role in the inflammatory response, with two distinct populations having opposing effects. Unruptured IA exhibits a balance between pro-inflammatory M1 and anti-inflammatory M2 macrophages, while ruptured IA shows an increase in M1 cells, suggesting a role for M1/M2 imbalance in rupture [22]. Early IA formation involves SMC migration to the intima and proliferation due to endothelial injury, leading to intimal hyperplasia. Subsequently, SMCs undergo a phenotypic switch from contractile to dedifferentiated, promoting inflammation and matrix degradation [23]. The inflammatory response further damages the arterial wall through the activation of molecules like tumor necrosis factor-alpha (TNF-α), monocyte chemoattractant protein-1 (MCP-1), interleukin-1β (IL-1β), Nuclear factor kappa-B (NF-κB), matrix metalloproteinases (MMPs), cyclooxygenase-1 (COX-1), and cyclooxygenase-2 (COX-2) [8]. The influx of blood exposes structurally weak arteries to high wall shear forces, leading to the formation of aneurysmal sacs. The aneurysmal sac grows until a balance is reached between ongoing vascular wall repair and extracellular matrix degradation [12] (Figure 1). The precise molecular mechanisms and inflammatory mediators leading to aneurysm rupture remain uncertain.

## 3. Research Progress on Biomarkers for Intracranial Aneurysms

Biomarkers are molecules that can indicate the presence or severity of a disease. In recent years, the development of “omics” technologies (genomics, transcriptomics, proteomics, and metabolomics) has accelerated the discovery of potential biomarkers for IA [24]. These biomarkers include nucleic acids, proteins, sugars, lipids, small metabolites, and extracellular vesicles (exosomes). For example, miR-29a was identified as a potential biomarker for early detection of IA and a prognostic indicator for the course of the disease by Wang et al. [25]. Similarly, Kao et al. suggested that plasma levels of interleukin-6 (IL-6) may be an early predictor of outcomes in patients with ruptured IA [26].

**Figure 2:**
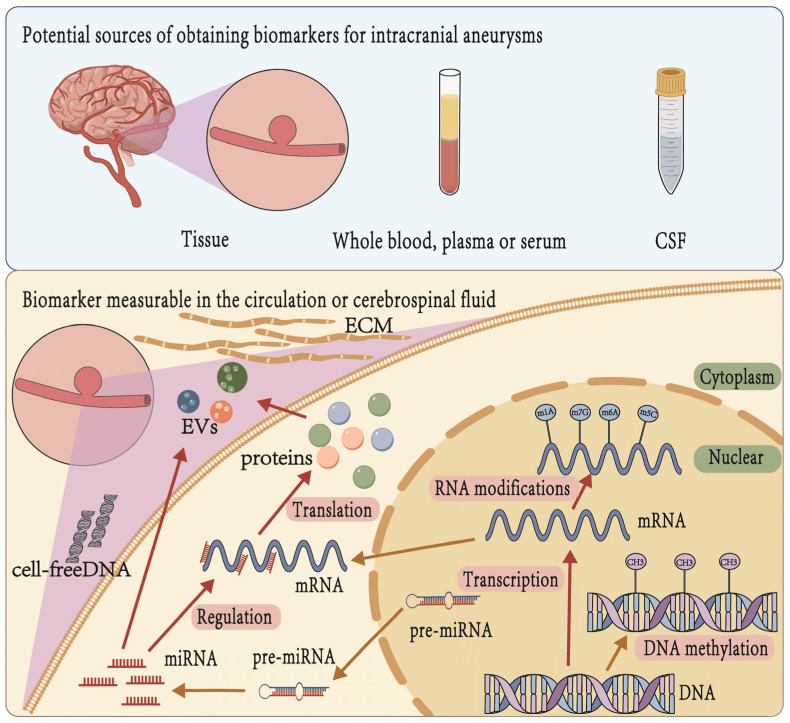
Schematic diagram of potential sources and measurable types of biomarkers for intracranial aneurysms [27,28,29]. Cerebrovascular tissue, blood, and cerebrospinal fluid are potential sources of biomarkers [30,31,32].

This review summarizes the potential biomarkers for IA identified in recent years, including extracellular vesicles, circulating nucleic acids (DNA, RNA, and microRNA [miRNA]), and proteins (Figure 2). There is considerable promise in these biomarkers for the early detection of IA, allowing for interventions before aneurysm rupture. Early diagnosis can significantly improve treatment success rates, reduce mortality rates, and enhance patient quality of life.

## 4. Sources of Biomarkers for IA

Biomarkers for IA can be categorized based on their source into tissue-derived, blood-derived, and cerebrospinal fluid (CSF)-derived biomarkers (Figure 2). Tissue biomarkers are primarily derived from affected organs or pathological sites and are obtained through biopsy, surgical resection, or autopsy. These biomarkers typically include specific gene mutations, changes in RNA expression levels, proteomic characteristics, and alterations in the tissue microenvironment [30]. In IA research, tissue biomarkers can be used to analyze structural changes in the vascular wall, the expression of inflammatory factors, and the characteristics of extracellular matrix (ECM) remodeling [33]. However, the acquisition of tissue samples is relatively invasive, which limits their clinical application. Blood is one of the most important sources of biomarkers, offering advantages such as ease of collection, repeatability of detection, and strong potential for clinical translation. Blood biomarkers primarily include circulating RNAs (such as miRNA and lncRNA), cell-free DNA (cfDNA), proteins (including inflammatory factors, enzymes, and receptors), and extracellular vesicles (EVs). In intracranial aneurysm research, blood biomarkers are widely used to assess inflammation status, oxidative stress levels, and changes in gene expression [31]. Additionally, due to the regulatory role of the blood–brain barrier, changes in specific biomarkers in the blood may indirectly reflect the progression of brain diseases [34]. CSF is directly connected to the central nervous system (CNS) and serves as a crucial carrier of biomarkers for neurological diseases [35]. CSF biomarkers include proteins (such as neuroinjury-related proteins), non-coding RNAs, metabolic products, and extracellular vesicles. Since CSF more directly reflects the physiological and pathological changes in intracranial lesions compared with blood, it exhibits higher sensitivity in cerebrovascular disease research [32]. In patients with intracranial aneurysms, inflammatory factors, extracellular matrix degradation products, and neuron injury-related molecules in CSF may serve as important diagnostic and prognostic biomarkers [36].

## 5. DNA

Genetic variations and epigenetic abnormalities in DNA significantly impact IA formation and rupture. Roder et al. analyzed gene expression data from IA tissue samples, identifying seven genes (B-cell lymphoma-2 (*BCL2*), collagen type I alpha 2 chain (*COL1A2*), collagen type III alpha 1 chain (*COL3A1*), collagen type V alpha 2 chain (*COL5A2*), chemokine (*C-X-C motif*) ligand 12 (*CXCL12*), tissue inhibitor of metalloproteinase 4 (*TIMP4*), and Tenascin C (*TNC*)) potentially involved in the genetic basis of IA [37]. McColgan et al. conducted a meta-analysis that revealed associations between polymorphisms in the endothelial nitric oxide synthase (*eNOS*) and *IL-6* genes and ruptured/unruptured IA, while the IL-6 G174C polymorphism showed a protective effect [38]. Researchers recently found that the gene serine protease inhibitor clade A member 3 (*SERPINA3*), which encodes a serine protease inhibitor, increases the risk of aneurysmal subarachnoid hemorrhage in Polish people [39]. However, Liu et al. did not find an association between the rs4934 polymorphism of *SERPINA3* and sporadic IA in a Chinese population, highlighting potential ethnic variations [40]. GWASs have identified several SNPs associated with IA [41] (Table 1). Yasuno et al. demonstrated the association of SNPs within three intervals located at chromosomes 4q31.23, 12q22, and 20p12.1 with IA. The most significant locus, at 4q31.23, contains the endothelin receptor type A (*EDNRA*) gene, which may be involved in IA progression and rupture [42].

DNA methylation, an epigenetic modification involving the addition of a methyl group to DNA, can also influence disease susceptibility [43]. DNA methylation patterns are regulated by both genetic and environmental factors and play a crucial role in gene expression [27,44]. Disruptions in DNA methylation patterns have been linked to various diseases, including IA [27]. For example, Zhao et al. [45] suggested that the methylation level of mitogen-activated protein kinase 10 (*MAP3K10*) might serve as a predictive marker for IA risk, particularly in women. Similarly, Xu et al. [46] found glutathione S-transferase alpha 4 (*GSTA4*) mRNA expression and gender-specific DNA methylation in relation to IA. Zhou et al. [47] observed significantly higher levels of patatin-like phospholipase domain-containing protein 6 (*PNPLA6*) methylation in IA patients compared with the control group. Maimaiti et al.’s bioinformatics study [48] linked DNA methyltransferase 3 alpha (*DNMT3A*) to lower SAH and unruptured intracranial aneurysm (UIA) risks, whereas methyl-CpG-binding domain protein 2 (*MBD2*) was linked to higher UIA risks, highlighting the role that DNA methylation plays in the pathogenesis of IA.

**Table 1:** Potential IA nucleic acid biomarkers.

Reference	Biomarker	Sample Source	n	Time of Take	Methodology	IA vs. Control	RIA vs. UIA
Zhong et al. [49]	miR-205	human blood	91	pretreatment	qRT-PCR	↑	
Feng et al. [50]	miR-155-5p	rats	NA	pre/posttreatment	qRT-PCR	↑	
Yu et al. [51]	miR-31a-5p	rats	NA	pre/posttreatment	qRT-PCR	↓	
Xiong et al. [52]	miR-125a	human blood	50	pretreatment	qRT-PCR	↑	
Holcomb et al. [53]	miR-1	rabbit	6	posttreatment	Sequencing	↓	
	miR-9-5p	rabbit	6	posttreatment	Sequencing	↓	
	miR-204-5p	rabbit	6	posttreatment	Sequencing	↓	
	miR-10a-5p	rabbit	6	posttreatment	Sequencing	↑	
	miR-21-5p	rabbit	6	posttreatment	Sequencing	↑	
	miR-34a-5p	rabbit	6	posttreatment	Sequencing	↑	
	miR-146a-5p	rabbit	6	posttreatment	Sequencing	↑	
	miR-223-3p	rabbit	6	posttreatment	Sequencing	↑	
Jiang et al. [54]	miR-1	IA tissues	14	intraoperative	qRT-PCR	↓	
	miR-7-1-3p	IA tissues	14	intraoperative	qRT-PCR	↓	
	miR-23b-5p	IA tissues	14	intraoperative	qRT-PCR	↓	
	miR-23b-3p	IA tissues	14	intraoperative	qRT-PCR	↓	
	miR-24-1-5p	IA tissues	14	intraoperative	qRT-PCR	↓	
	miR-28-5p	IA tissues	14	intraoperative	qRT-PCR	↓	
	miR-28-3p	IA tissues	14	intraoperative	qRT-PCR	↓	
	miR-29b-2-5p	IA tissues	14	intraoperative	qRT-PCR	↓	
	miR-29c-5p	IA tissues	14	intraoperative	qRT-PCR	↓	
	miR-29c-3p	IA tissues	14	intraoperative	qRT-PCR	↓	
	miR-133a	IA tissues	14	intraoperative	qRT-PCR	↓	
	miR-133b	IA tissues	14	intraoperative	qRT-PCR	↓	
	miR-140-3p	IA tissues	14	intraoperative	qRT-PCR	↓	
	miR-143-5p	IA tissues	14	intraoperative	qRT-PCR	↓	
	miR-143-3p	IA tissues	14	intraoperative	qRT-PCR	↓	
	miR-145-5p	IA tissues	14	intraoperative	qRT-PCR	↓	
	miR-145-3p	IA tissues	14	intraoperative	qRT-PCR	↓	
	miR-455-5p	IA tissues	14	intraoperative	qRT-PCR	↓	
Zhao et al. [55]	miR-29a	human blood	24	pre/posttreatment	qRT-PCR	↑	
Zheng et al. [56]	miR-513b-5p	human serum	100	pretreatment	qRT-PCR	↓	↓
Fan et al. [57]	miR-331-3p	IA tissues	96	intraoperative	RT-qPCR	↓	
Wang et al. [25]	miR-29a	human plasma	165	pretreatment	qRT-PCR	↑	
Bekelis et al. [58]	miR-21	IA tissues	7	intraoperative	sequencing	↑	
	miR-143-5p	IA tissues	7	intraoperative	sequencing	↓	
	miR-145	IA tissues	7	intraoperative	sequencing	↓	
Lv et al. [59]	miR-136-5p	IA tissues	82	intraoperative	qRT-PCR	↓	
Cai et al. [60]	miR-92a	IA tissues	90	intraoperative	qRT-PCR	↓	
Liu et al. [61]	miR-29b	IA tissues	6	intraoperative	qRT-PCR	↓	
Guo et al. [62]	miR-23b-3p	IA tissues	32	intraoperative	qRT-PCR	↓	
Jin et al. [63]	miR-22	human plasma	24	pretreatment	PCA	↑	
	miR-671-5p	human plasma	24	pretreatment	PCA	↑	
	miR-720	human plasma	24	pretreatment	PCA	↑	
	miR-365	human plasma	24	pretreatment	PCA	↓	
	miR-498	human plasma	24	pretreatment	PCA	↑	
	miR-574	human plasma	24	pretreatment	PCA	↓	
	miR-106b	human plasma	24	pretreatment	PCA	↑	
	miR-21	human plasma	24	pretreatment	PCA	↑	
	miR-936	human plasma	24	pretreatment	PCA	↓	
Su et al. [64]	miR-132	human plasma	58	pretreatment	qRT-PCR	↑	
	miR-324	human plasma	58	pretreatment	qRT-PCR	↑	
Supriya et al. [65]	miR-26b	IA tissues	29	intraoperative	qRT-PCR	↓	
	miR-199a	IA tissues	29	intraoperative	qRT-PCR	↓	
	miR-497	IA tissues	29	intraoperative	qRT-PCR	↓	
	miR-365	IA tissues	29	intraoperative	qRT-PCR	↓	
Luo et al. [66]	miR-9	IA tissues	13	intraoperative	qRT-PCR	↑	
Yang et al. [67]	miR-144-5p	human plasma	84	pretreatment	qRT-PCR	↓	
Liao et al. [68]	miR-145-5p	human plasma	12	pretreatment	qRT-PCR	↑	
	miR-29a-3p	human plasma	12	pretreatment	qRT-PCR	↑	
Li et al. [69]	miR-16	human plasma	40	pretreatment	qRT-PCR	↑	
	miR-25	human plasma	40	pretreatment	qRT-PCR	↑	
Zou et al. [70]	miR-34a	human blood	20	intraoperative	qRT-PCR	↓	
Xu et al. [71]	miR-143	human serum	30	intraoperative	qRT-PCR	↓	
	miR-145	human serum	30	intraoperative	qRT-PCR	↓	
Yuan et al. [72]	miR-34a	human serum	20	intraoperative	qRT-PCR	↓	
Zhao et al. [45]	*Methyl MAP3K10*	human plasma	96	pretreatment	MIRA-seq	↓	
Xu et al. [46]	*Methyl GSTA4*	human blood	44	pretreatment	MIRA-seq	↓	
Zhou et al. [47]	*Methyl PNPLA6*	human plasma	96	pretreatment	MIRA-seq	↑	
Roder et al. [37]	*BCL2*	/	30	pretreatment	MIRA-seq	↓	
	*COL1A2*	/	30	pretreatment	MIRA-seq	↑	
	*COL3A1*	/	30	pretreatment	MIRA-seq	↑	
	*COL5A2*	/	30	pretreatment	MIRA-seq	↑	
	*CXCL12*	/	30	pretreatment	MIRA-seq	↓	
	*TIMP4*	/	30	pretreatment	MIRA-seq	↓	
	*TNC*	/	30	pretreatment	MIRA-seq	↓	
Maimaiti et al. [48]	*DNMT3A*	/	156	pretreatment	MIRA-seq	↓	
	*MBD2*	/	156	pretreatment	MIRA-seq	↑	

Abbreviations: IA, intracranial aneurysm; UIA, unruptured intracranial aneurysm; RIA, ruptured intracranial aneurysm; qRT-PCR, quantitative real-time polymerase chain reaction; PCA, principal component analyzed; MIRA-seq, methylated-pyrosequencing; Methyl, methylation; *MAP3K10*, mitogen-activated protein kinase 10; *GSTA4*, glutathione S-transferase alpha 4; *PNPLA6*, patatin-like phospholipase domain-containing protein 6; *BCL2*, B-cell lymphoma-2; *COL1A2*, collagen type I alpha 2 chain; *COL3A1*, collagen type III alpha 1 chain; *COL5A2*, collagen type V alpha 2 chain; *CXCL12*, chemokine (*C-X-C motif*) ligand 12; *TIMP4*, tissue inhibitor of metalloproteinase 4; *TNC*, Tenascin C; *DNMT3A*, DNA methyltransferase 3 alpha; *MBD2*, methyl-CpG-binding domain protein 2; ↑, upregulate; ↓, downregulate.

## 6. RNA

**Figure 3:**
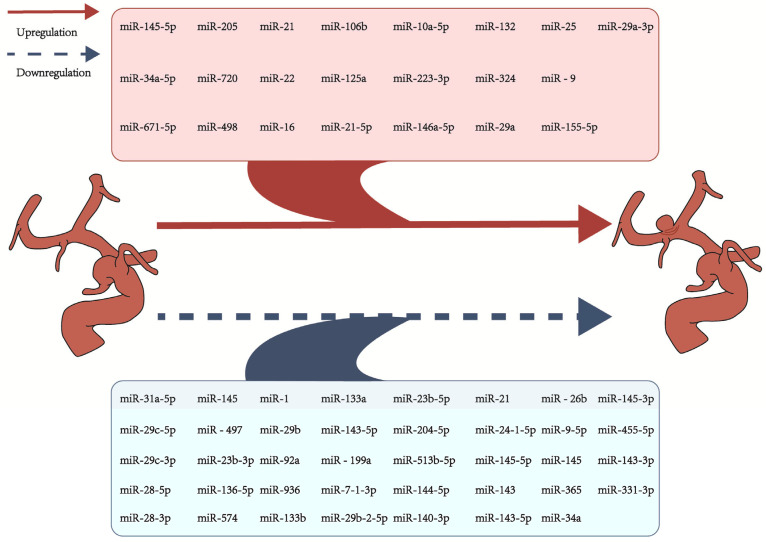
MiRNAs upregulated and downregulated during the formation of intracranial aneurysms [25,49,50,51,52,55,56,58,60,61,63,64,65,66,68,69,70,71,72].

MicroRNAs (miRNAs) are small, non-coding RNAs, typically consisting of approximately 22 nucleotides, that regulate gene expression through transcription or translation interference [28]. They play crucial roles in various physiological processes, including cell cycle progression, stem cell differentiation, organ growth, and signal transduction [73]. miRNAs are also involved in tumorigenesis, regulation of oxidative stress, DNA damage, and the organismal response to radiation [74]. According to several studies, miRNAs play a crucial role in IA formation and rupture (Figure 3) (Table 1). Bekelis et al. [58] identified differentially expressed genes by analyzing tissue specimens from IA and control subjects. Matrix metalloproteinase-13 (*MMP-13*) and various collagen genes [ collagen type I alpha 1 chain (*COL1A1*), collagen type V alpha 1 chain (*COL5A1*) and alpha 2 chain (*COL5A2*) ], and miR-21 showed the greatest upregulation, while miR-143-5p showed the greatest downregulation. Jiang et al. [54] used microarrays to compare miRNA expression between IA tissue and normal tissue. They identified 18 significantly differentially expressed miRNAs, including miR-1, miR-24-1-5p, miR-29b-2-5p, miR-7-1-3p, miR-29c-5p, miR-29c-3p, miR-133a, miR-133b, miR-23b-3p, miR-140-3p, miR-143-5p, miR-143-3p, miR-23b-3p, miR-145-5p, miR-145-3p, miR-455-5p, and others. Functional analysis revealed that the predicted target genes of these miRNAs were associated with processes such as macrophage migration, monocyte proliferation, and smooth muscle cell migration, which are important in IA development. Fan et al. [57] analyzed the IA microarray GSE75436 from the gene expression omnibus (GEO) database, which included normal and IA samples. Their analysis revealed significantly decreased miR-331-3p expression in IA. They proposed that miR-331-3p plays a role in IA by regulating the VSMC phenotype and inhibiting TNF-α and CD14 through the NF-κB signaling pathway, indicating its potential as a therapeutic target and prognostic marker. Compared with microarrays, next-generation RNA sequencing (RNA-seq) offers higher specificity, sensitivity, and a wider dynamic range. It can also detect novel transcripts and isoforms [75]. Zhong et al. found that miRNA-205 levels in the blood of IA patients were significantly higher than those in the healthy control group, suggesting its potential as a diagnostic biomarker [49]. Conversely, Xiong et al. [52] observed elevated levels of miR-125a in the blood of IA patients compared with the control group, potentially increasing the risk of IA rebleeding. Interestingly, miR-125a is usually downregulated in certain cancers, suggesting complex regulatory mechanisms. Holcomb et al. [53] identified miRNAs that were upregulated (miR-10a-5p, miR-21-5p, miR-34a-5p, miR-146a-5p, and miR-146a-5p) and downregulated (miR-1, miR-9-5p, and miR-204-5p) in arterial aneurysm tissues. Pathway analysis suggested that some upregulated miRNAs might promote inflammation, while the downregulated miRNAs might inhibit cell migration and coagulation, affecting aneurysm development. Zhao et al. [55] found that miR-29a is significantly upregulated in IA tissues and promotes apoptosis, leading to increased DNA damage and mitochondrial release. It may also reduce the expression of myeloid leukemia 1 (*Mcl-1*) and increase the expression of caspase-3 and cytochrome c, promoting the activation of the apoptosis pathway. These findings suggest that miR-29a could be a novel target for IA prevention and treatment. Consistent with these results, Wang et al. [25] also found miR-29a to be a potential biomarker for IA. Zheng et al. [56] found that there was a decrease in miR-513b-5p expression in both unruptured and ruptured IA patients compared with the healthy control group. Their experiments suggest that miR-513b-5p targets *COL1A2* and *COL1A1* to inhibit smooth muscle cell proliferation and promotes cell death and apoptosis. This aligns with previous studies suggesting an anti-proliferative and pro-apoptotic role for miR-513b-5p [76]. Yuan et al. [72] observed that serum levels of miR-34a were significantly decreased in IA patients compared with the control group, suggesting a potential role for miR-34a in IA formation and progression and potentially serving as a biomarker for early detection. Yang et al. found that serum levels of extracellular vesicle miR-144-5p were significantly lower in IA patients compared with healthy individuals [67]. Conversely, Liao et al. reported elevated levels of miR-145-5p and miR-29a-3p in circulating EVs from IA patients compared with controls, suggesting their potential use for monitoring IA formation and rupture [68]. However, Xu et al. [71] observed downregulation of miR-143/145 in the serum of IA patients, contradicting Liao et al.’s findings [68]. This discrepancy highlights the complexity of miRNA regulation in IA and the need for further investigation. MiR-145 is known to regulate VSMC phenotype and proliferation, potentially impacting IA pathogenesis [71]. In vitro experiments suggest that miR-143/145 may counteract the regulatory effects of Kruppel-like factor 5 (*KLF5*) in VSMCs, influencing cell proliferation and contraction in IA. The differing results observed by Liao et al. [68] and Xu et al. [71] could be due to compensatory mechanisms at play [71]. Li et al. identified miR-16 and miR-25 as potential circulating miRNA biomarkers for IA using microarray profiling and validation with quantitative real-time polymerase chain reaction (qRT-PCR) [69]. Feng et al. proposed that tumor-associated macrophage (*TAM*)-derived exosomal miR-155-5p promotes IA formation by antagonizing gremlin 1 (*GREM1*), a secreted bone morphogenetic protein antagonist. This suggests that miR-155-5p may serve as a therapeutic target and a biomarker for IA [50].

RNA modifications, including N1-methyladenosine (m1A), N6-methyladenosine (m6A), m7G, 2′-O-methylation, 5-methylcytosine (m5C), and ac4C RNA acetylation, represent another layer of epigenetic regulation influencing RNA metabolism and processing [77]. m6A is considered the most abundant form. m6A modification occurs on adenosine within the RRACH sequence and is regulated by “Writer”, “Eraser”, and “Reader” complexes [78]. Mounting evidence suggests the crucial role of m6A methylation in various diseases, including cancer [79]. However, limited research has been conducted on m6A methylation in IA. Maimaiti et al. [80] analyzed 60 samples (44 IA, 16 normal) and identified 8 m6A markers (*IGFBP2, IGFBP1, IGF2BP2, YTHDF3, ALKBH5, RBM15B, LRPPRC,* and *ELAVL1*) that correlated significantly with IA, suggesting their potential as prognostic biomarkers. Li et al. [81] analyzed three datasets and found nine differentially expressed m6A regulators (insulin-like growth factor 2 mRNA-binding protein 1 (*IGF2BP1*), insulin-like growth factor 2 mRNA-binding protein 3 (*IGF2BP3*), zinc finger protein 217 (*ZNF217*), YT521-B homology domain family member 2 (*YTHDF2*), YT521-B homology domain family member 3 (*YTHDF3*), YT521-B homology domain-containing protein 1 (*YTHDC1*), fat mass and obesity-associated protein (*FTO*), RNA-binding motif protein 15 (*RBM15*), and leucine-rich pentatricopeptide repeat containing (*LRPPRC*)) between the control and IA groups, also indicating their potential as biomarkers. However, these studies have limitations. The small sample sizes may lead to bias, and the validation of m6A regulatory factor expression was limited to blood samples and did not encompass IA tissues. Additionally, specific m6A sites have not been investigated using techniques like MeRIP-seq and MeRIP-qPCR. These techniques could help identify the target genes of m6A regulatory factors that play a role in IA development.

## 7. Proteins

### 7.1. Inflammation-Related Proteins

Inflammation, a natural response to injury characterized by swelling, redness, and increased white blood cell infiltration, plays a dual role in the context of IA. While it promotes initial tissue repair, chronic inflammation can lead to detrimental effects [82]. The development of IA involves distinct stages: endothelial injury and elastic layer degradation, recruitment and infiltration of inflammatory cells, and chronic vascular wall remodeling [29]. Inflammatory cell infiltration is a crucial step, as these cells secrete inflammatory cytokines that regulate, activate, and promote the proliferation, migration, and apoptosis of immune and endothelial cells. Additionally, they release matrix-degrading collagenases and proteases, which contribute to aneurysm formation and progression [29]. This section highlights the role of key inflammatory-related proteins in IA (Table 2).

Monocyte chemoattractant protein-1 (MCP-1), a chemokine expressed by microglia, neurons, astrocytes, and endothelial cells, serves as a critical player in monocyte recruitment [83]. It promotes monocyte aggregation in damaged areas by regulating adhesion molecules and modulating membrane-binding activity. MCP-1 also induces the release of molecules that degrade elastic and collagen fibers in the arterial wall (e.g., MMPs), leading to aneurysm formation [83]. Under high shear stress conditions within the aneurysmal wall, endothelial cells express MCP-1, facilitating early-stage macrophage infiltration [12]. Studies have shown that MCP-1 levels are elevated in ruptured IAs compared with unruptured IAs, while miR-493, a competitive endogenous RNA of AC007362 and a direct target gene of miR-493, exhibits an inverse relationship with MCP-1 expression [31,84]. These findings suggest that MCP-1 may serve as a biomarker for IA rupture. Additionally, in IA patients, MCP-1 levels were observed to be elevated in comparison with those in healthy controls, with further increases observed in patients with ruptured or multiple aneurysms [82,85]. These results highlight the critical role of MCP-1 in IA formation, development, and rupture. Liu et al. [86] observed a significant increase in MCP-1 expression in an IA model, suggesting continuous production by SMCs and endothelial cells, potentially contributing to a sustained inflammatory response. Notably, MCP-1 expression may serve as an early indicator for IA detection, as its mRNA levels rapidly peak within a week after induction [86].Tumor necrosis factor-alpha (TNF-α) is a pro-inflammatory cytokine involved in the inflammatory response cascade, and it plays a role in initiating cell apoptosis signaling pathways [87]. Produced by macrophages, endothelial cells, and SMCs within IA, TNF-α contributes to pro-inflammatory changes [88]. It acts as a pro-inflammatory factor in IAs, inducing macrophage polarization and promoting atherosclerosis development [89]. TNF-α exerts its pro-atherosclerotic effects through various mechanisms, including disrupting endothelial junctions (leading to dysfunction and instability) and facilitating leukocyte migration by stimulating adhesion molecules, particularly Vascular Cell Adhesion Molecule 1 (VCAM-1) [87,88]. Kanematsu et al. [90] demonstrated that TNF-α directly activates genes in cerebral vascular SMCs associated with IA formation, including matrix metalloproteinase-3(MMP-3), matrix metalloproteinase-9(MMP-9), MCP-1, VCAM-1, and IL-1β, all involved in pro-inflammatory responses and matrix remodeling. Fan et al. [57] revealed increased expression of TNF-α, ICAM-1, MCP-1, IL-6, matrix metalloproteinase-2 (MMP-2), and MMP-9 in IA patient vessels compared with controls. These findings support the role of TNF-α in promoting inflammation and matrix remodeling in IA. Similar to MCP-1, TNF-α levels are elevated in both unruptured and ruptured IAs compared with controls, with higher levels observed in ruptured IAs, suggesting its potential role in promoting both formation and rupture [85]. Starke et al. reported increased TNF-α expression in a mouse model of IA, with further elevation in ruptured aneurysms [91]. Additionally, TNF-α gene knockout mice and mice pretreated with TNF-α inhibitors displayed a decreased incidence of IA formation and rupture, suggesting its involvement in these processes [91]. However, the precise role of TNF-α in aneurysm progression and rupture remains to be elucidated. Jayaraman et al. observed increased expression of TNF-α and its downstream apoptotic target fatty acid synthase (FAS) in IA, suggesting its role in promoting apoptosis and inflammation within the aneurysm wall [92]. TNF-α may also serve as a potential biomarker for IA formation and rupture.

**Table 2:** Potential IA protein biomarkers.

Reference	Biomarker	Sample Source	n	Time of Take	Methodology	IA vs. Control	RIA vs. UIA
Xiong et al. [52]	ET-1	blood	50	pretreatment	WB	↑	
Lv et al. [59]	KDM1A	IA tissues	82	intraoperative	WB	↑	
Cai et al. [60]	HDAC9	IA tissues	90	intraoperative	WB	↑	
	BCL2L11	IA tissues	90	intraoperative	WB	↑	
Fan et al. [57]	TNF-α	IA tissues	96	intraoperative	WB	↑	
	CD14	IA tissues	96	intraoperative	WB	↑	
Zheng et al. [56]	COL1A1	serum	100	pretreatment	WB	↑	↑
	COL1A2	serum	100	pretreatment	WB	↑	↑
	TNF-α	serum	100	pretreatment	WB	↑	↑
	IL-1β	serum	100	pretreatment	WB	↑	↑
	MMP-2	serum	100	pretreatment	WB	↑	↑
	MMP-3	serum	100	pretreatment	WB	↑	↑
	MMP-9	serum	100	pretreatment	WB	↑	↑
Guo et al. [62]	PTEN	IA tissues	32	intraoperative	WB	↑	
Xu et al. [46]	GSTA4	serum	44	pretreatment	WB	↑	
Zhou et al. [47]	PNPLA6	serum	96	pretreatment	WB	↓	
Zhang et al. [82]	IL-1β	blood	66	pretreatment	Bio-Plex protein array systems	↑	
	MCP-1	blood	66	pretreatment	Bio-Plex protein array systems	↑	
	TNF-α	blood	66	pretreatment	Bio-Plex protein array systems	↑	
Yamaguchi et al. [93]	MMP-3	IA tissues	24	pre/intraoperative	WB		↑
	IL-1β	IA tissues	24	pre/intraoperative	WB		↑
Zou et al. [70]	MMP-2	IA tissues	20	pre/intraoperative	WB	↑	
Kamińska et al. [94]	MCP-1	CSF	25	pretreatment	ELISA	↑	
	IL-8	CSF	25	pretreatment	ELISA	↑	
Lai et al. [85]	TNF-α	serum	108	pretreatment	ELISA	↑	↑
	MCP-1	serum	108	pretreatment	ELISA	↑	↑
	IL-1β	serum	108	pretreatment	ELISA	↑	↑
	IL-6	serum	108	pretreatment	ELISA	↑	↑
	NF-κB p65	serum	108	pretreatment	ELISA	↑	
Kamińska et al. [95]	NF-κB p65	CSF, serum	25	pretreatment	ELISA	↓	
	CXCL1	CSF, serum	25	pretreatment	ELISA	↑	
	CXCR2	CSF, serum	25	pretreatment	ELISA	↑	
Aoki et al. [96]	COX-2	mice IA tissues	5	pretreatment	WB	↑	
	mPGES1	mice IA tissues	5	pretreatment	WB	↑	
Sun et al. [97]	NOX4	IA tissues	27	pretreatment	WB	↑	
	p22phox	IA tissues	27	pretreatment	WB	↑	
	p47phox	IA tissues	27	pretreatment	WB	↑	
	TRPC6	IA tissues	27	pretreatment	WB	↑	
	CN	IA tissues	27	pretreatment	WB	↑	
	NFATC1	IA tissues	27	pretreatment	WB	↑	
	MMP-2	IA tissues	27	pretreatment	WB	↑	
	MCP-1	IA tissues	27	pretreatment	WB	↑	
Aoki et al. [98]	procollagen type I	IA tissues	6	pretreatment	WB	↓	
	procollagen type III	IA tissues	6	pretreatment	WB	↓	

Abbreviations: ET-1, endothelin-1; KDM1A, lysine-specific demethylase 1; HDAC9, Histone deacetylase 9; BCL2L11, BCL2 (B-cell lymphoma-2)-like protein 11; PTEN, phosphatase and tensin homolog; GSTA4, glutathione S-transferase alpha 4; PNPLA6, patatin-like phospholipase domain-containing protein 6; NF-κB p65, Nuclear factor kappa-B p65 subunit; CXCL1, C-X-C motif ligand 1; CXCR2,C-X-C Motif Chemokine Receptor 2; TRPC6,The transient receptor potential canonical 6; CN, Calcineurin; NFATC1, nuclear factor of activated T cell; TNF-α, tumor necrosis factor-α; CD14, cluster of differentiation 14; COL1A1, collagen type I alpha 1 chain; COL1A2, collagen type I alpha 2 chain; IL-1β, interleukin-1β; MMP-2, Matrix Metallopeptidase 2; MMP-3, Matrix Metallopeptidase 3; MMP-9, Matrix Metallopeptidase 9; MCP-1, monocyte chemotactic protein-1; IL-8, interleukin-8; IL-6, interleukin-6; COX-2, Cyclooxygenase-2; mPGES1, microsomal prostaglandin E synthase-1; NOX4, NADPH oxidase 4; p22phox, Cytochrome b-245, alpha polypeptide; IA, intracranial aneurysm; UIA, unruptured intracranial aneurysm; RIA, ruptured intracranial aneurysm; CSF, cerebrospinal fluid; WB, Western blotting; ELISA, Enzyme-linked immunosorbent assay; p47phox, Cyclin-dependent kinase inhibitor p27; ↑, upregulate; ↓, downregulate.

IL-1β is a pro-inflammatory cytokine produced in its precursor form by activated macrophages and processed into its active form by caspase 1 (CASP1/ICE). It is produced by various cell types, including monocytes, macrophages, T cells, B cells, dendritic cells, endothelial cells, and VSMCs [99]. IL-1β plays a crucial role in inflammatory responses, influencing cell proliferation, differentiation, and apoptosis. Studies have shown that IL-1β contributes to inflammatory pain hypersensitivity by inducing cyclooxygenase-2 (PTGS2/COX2) in the central nervous system (CNS) [100]. Animal models of IAs and abdominal aortic aneurysms (AAAs) suggest that IL-1β upregulation promotes aneurysm progression by regulating collagen biosynthesis in the aneurysm wall [101]. In a study by Moriwaki, T. et al., IL-1β^−/−^ mice displayed a significantly lower number of late-stage aneurysms compared with wild-type mice, with a notable reduction in cellular apoptosis in the aneurysm tissue [102]. These findings suggest a protective role for IL-1β deficiency against late-stage aneurysm development. Zheng et al. reported there was a notable rise in IL-1β mRNA expression among IA patients., with further elevation observed in ruptured compared with unruptured IAs [56]. They proposed that necrotic VSMCs in aneurysms can release IL-1β, inducing IL-6 production, thereby promoting vascular inflammation and accelerating aneurysm progression. Additionally, Zhang et al. [82] and Lai et al. [85] found elevated IL-1β levels in IA patients, suggesting its importance in aneurysm development and rupture. Liu et al. [103] investigated the correlation between arterial wall enhancement and cellular apoptosis. They found that patients with unruptured IAs showing arterial wall enhancement had upregulated pro-inflammatory cytokines like IL-1β and downregulated anti-inflammatory cytokines like IL-1ra. This suggests that the ratio of IL-1β to IL-1ra might serve as a potential biomarker for predicting aneurysm rupture or growth.

NF-κB is a transcription factor that regulates various inflammation-related genes in response to external stimuli. The NF-κB protein family consists of two main classes: class I (including NF-κB1/p105 and NF-κB2/p100) and class II (including RelA/p65, RelB, and c-Rel) [104]. NF-κB signaling involves two interconnected pathways, the canonical pathway and the non-canonical pathway, which differ in their components and functions [104]. In mammals, NF-κB forms homodimers or heterodimers from five subunits: p50, p52, RelA (p65), RelB, and c-Rel. Aoki et al. [105] demonstrated the crucial role of the p50 subunit in initiating IA formation. They found that p50−/− mice exhibited reduced expression of MCP-1 and VCAM-1, as well as decreased macrophage infiltration in the cerebral artery wall, compared with p50+/+ mice after aneurysm induction. This suggests that p50 plays a key role in macrophage aggregation and potentially in degenerative changes in the aneurysm wall through transcriptional regulation. Smoking, alcohol abuse, obesity, and oxidative stress are potential risk factors for IA formation and rupture. These factors can activate NF-κB, leading to increased expression of pro-inflammatory markers and MMPs [106]. In its inactive state, NF-κB resides in the cytoplasm. Upon stimulation, it becomes activated, translocates to the nucleus via nuclear localization sequences, and initiates the regulation of target genes [107]. Liu et al. [86] observed strong nuclear expression of chemokines, adhesion molecules, MMPs, and other factors in aneurysmal cells, indicating NF-κB activation. Activated NF-κB promotes the expression of genes encoding chemokines, adhesion molecules, MMPs, etc., which directly influence the activation, proliferation, migration, and secretion processes of relevant cells [108]. These findings suggest that NF-κB plays a critical role in aneurysmal wall inflammation and the vascular pathology of IA. Interestingly, Lai et al. [85] detected increased NF-κB p65 protein expression in both unruptured and ruptured IA compared with normal vessels, suggesting its potential role as a risk factor. However, Kamińska et al. [95] reported lower NF-κB p65 concentrations in the serum and cerebrospinal fluid (CSF) of unruptured IA patients compared with controls, suggesting potential increased activation within the central nervous system. These findings suggest that NF-κB plays a complex role in IA, potentially influencing both initiation and progression. Further research is needed to elucidate the precise contribution of different NF-κB subunits and pathways at various stages of IA development.

IL-6, a multifunctional cytokine crucial for host defense, exerts distinct effects in the nervous system through classical and trans-signaling pathways [109]. Both pathways involve binding to either membrane-bound or soluble IL-6 receptors, which activate JAK/STAT and MAPK pathways, ultimately leading to gene transcription and protein expression [110,111]. The classical pathway, primarily mediated by membrane-bound receptors, has neuroprotective effects. Upon binding to glycoprotein 130 (gp130), IL-6 activates the JAK/STAT and MAPK pathways, promoting cell growth, survival, and gene expression [110,111]. This pathway additionally results in the generation of cytokine signaling suppressor 3 (SOCS3), an IL-6 signaling negative regulator, creating a feedback loop to limit further IL-6 activity [112]. Conversely, the trans-signaling pathway, involving soluble receptors, promotes inflammation. Soluble IL-6 receptors bind to gp130 on endothelial cells, activating similar JAK/STAT and MAPK pathways but in a wider range of cell types. This pathway also inhibits SOCS3, leading to sustained IL-6 signaling and increased production of inflammatory cytokines [112,113]. The role of IL-6 in IA development and progression remains to be fully elucidated. Animal studies suggest a potential link between IL-6 and IA formation. Reduced cytokine production, including IL-6, in macrophages following dipeptidyl peptidase-4 (DPP-4) inhibition correlated with smaller IA size in a rat model [114]. Clinical studies have explored IL-6 levels in IA patients. Wajima et al. [115] observed higher IL-6 expression in IA tissues compared with controls, with potentially higher levels in women. Kamińska et al. [116] reported an elevated IL-6 ratio (CSF to serum concentration) in unruptured IA patients, suggesting potential central nervous system involvement, especially in those with multiple aneurysms. Additionally, Kao et al. [26] identified elevated blood IL-6 within the first three days of IA rupture as an independent predictor of poor clinical outcomes. Interestingly, animal models of SAH, a complication of ruptured IA, suggest that IL-6 may contribute to vasospasm, a narrowing of blood vessels that can worsen neurological deficits [117]. These discoveries imply that IL-6 might have a complex function in various aspects of IA, potentially influencing the formation, growth, rupture, and post-rupture complications. Further research is needed to determine the precise role of IL-6 and its signaling pathways in IA pathogenesis.

### 7.2. Oxidative Stress-Related Proteins

**Figure 4:**
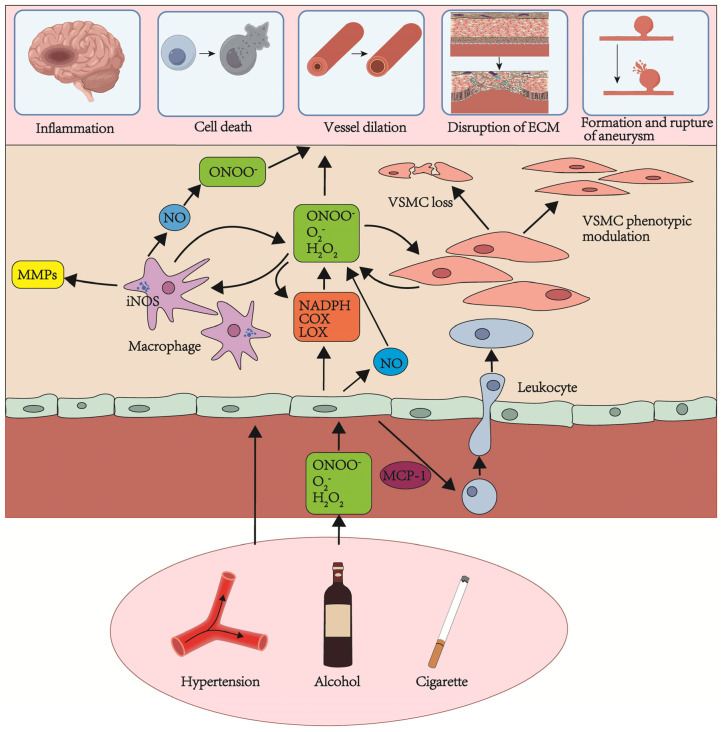
Potential sources and roles of oxidative stress in the pathogenesis of intracranial aneurysms [118,119,120,122].

Oxidative stress, resulting from an imbalance between the generation and removal of free radicals, plays a pivotal role in the pathogenesis of IA. This imbalance leads to DNA damage, cellular toxicity, and apoptosis [118]. In the pathogenesis of IA, crucial pathways of oxidative stress involve atherosclerosis, hemodynamic stress, endothelial dysfunction, VSMC phenotype modulation, vascular remodeling, and apoptotic cell death. These pathways promote the generation of reactive oxygen species (ROS) such as superoxide (O_2_^•−^), hydrogen peroxide (H_2_O_2_), and peroxynitrite (ONOO^•−^) [119]. Recognized risk factors contributing to ROS production include cigarette smoke, alcohol, and hypertension (Figure 4). Smoking is a primary risk factor for IA formation and rupture, with approximately 80% of SAH patients reporting a significant smoking history [120]. Cigarette smoke is a major source of free radicals, activating Nicotinamide Adenine Dinucleotide Phosphate (NADPH) and generating O_2_^•−^ and H_2_O_2_. These may originate directly from gas/tar phases, activated white blood cells, or endogenous sources of ROS, such as xanthine oxidase, uncoupled eNOS, and the mitochondrial electron transport chain [121]. Alcohol is widely recognized as another significant risk factor for aneurysm rupture, with its effects potentially mediated through free radicals. However, further extensive research is needed to determine whether alcohol-induced oxidative stress directly leads to aneurysmal changes in cerebral vasculature [122]. Hypertension has been demonstrated as another risk factor for the formation and rupture of aneurysms [122]. Evidence suggests that O_2_^•−^, H_2_O_2_, and ONOO^•−^ generated through NADPH oxidase and uncoupled eNOS may play a crucial role in hypertension pathogenesis, although various other factors may also be involved [123]. The potential sources of oxidative stress in IA formation and rupture are numerous. The main enzymatic sources of ROS in the cerebrovascular system include COX, lipoxygenases (LOXs), and NADPH oxidase [124]. NOS is another potential source of free radicals in the cerebral circulation [125]. Oxidative stress-related proteins play an indispensable role in the process of IA formation and rupture (Table 2).

Cyclooxygenase-2 (COX-2), an inducible enzyme unlike the stable COX-1, plays a significant role in IA development. Studies have shown increased COX-2 expression in IA tissues compared with healthy controls [96]. This upregulation is likely due to the presence of unstable sequences in the COX-2 gene (Ptgs-2), making it readily activated during inflammatory processes [126,127]. A key function of COX-2 is the conversion of arachidonic acid to prostaglandin G2 (PGG2), a precursor for various inflammatory mediators. In the context of IA, the prostaglandin E2 (PGE2) derived from COX-2 activity appears to be particularly crucial [128]. PGE2 signaling through the EP2 receptor activates NF-κB, a transcription factor promoting further COX-2 expression. This positive feedback loop involving COX-2, PGE2, and NF-κB is believed to be a key driver of inflammation in the IA wall [96]. Therefore, targeting the COX-2 pathway may hold promise for therapeutic strategies aimed at preventing or mitigating IA formation.

Typical NADPH oxidase (NOX) is primarily expressed in polymorphonuclear neutrophils (PMNs), playing a central role in host defense and participating in bacterial killing by PMN. The enzyme consists of two membrane-bound subunits (gp91phox and p22phox) and at least three cytosolic subunits [129]. NOX is widely distributed in the vasculature, including endothelial cells, VSMCs, and adventitial cells of cerebral arteries, exhibiting active functionality. Various factors, including cytokines and mechanical stress, can induce vascular NOX to produce superoxide and hydrogen peroxide, contributing to oxidative stress in IA [130]. Tamura et al. [131] demonstrated in rat experiments that estrogen may mediate the expression of NADPH oxidase in aneurysms, providing a potential link between oxidative stress and the increased incidence of aneurysms in women. In VSMCs, the isoform NOX4 of NOX is the predominant form [132]. Studies have shown that ROS derived from NOX4 can induce phenotypic transition of human brain vascular smooth muscle cells (HBVSMCs) and promote the formation of IAs [133]. Sun et al. [97] found that the expression of NOX4 increased in IA patients, and the TRPC6-NFATC1 signaling was activated in IA patients. Their study demonstrated that interruption of TRPC6-NFATC1 signaling significantly downregulated NOX4 expression in IA and attenuated VSMC phenotypic transition, suggesting that TRPC6-NFATC1 signaling could be an important therapeutic target for treating IA. Intracellular Ca2+ imbalance mediated by transient receptor potential-6 (TRPC6) may also participate in the regulation of NOX4 and the VSMC phenotypic switch in the pathogenesis of IA.

The nitric oxide synthase (NOS) enzyme family consists of eNOS, located in endothelial cells; inducible NOS (iNOS), located in macrophages and VSMCs; and neuronal NOS (nNOS), located in neurons. NOS primarily synthesizes nitric oxide (NO), a hydrophobic molecule with a short half-life, which easily diffuses through neighboring cell membranes, exerting transient effects locally [134]. In vascular physiology, nitric oxide plays various crucial roles, including regulating vascular tone, inhibiting SMC proliferation, suppressing pro-inflammatory mediators, and maintaining the proper function and integrity of endothelial cells (ECs) [135]. Studies have found that while eNOS and nNOS have protective effects on IA, under abnormal conditions such as mechanical injury and inflammation, macrophages and VSMCs express iNOS [136]. ROS and inflammatory mediators (such as Interferon-gamma (IFN-γ), TNF-a, and IL-1b) increase the expression of iNOS, which is associated with the cytotoxic effects of NO. iNOS also impairs the activity of eNOS and nNOS through a negative feedback loop involving NO [119]. eNOS is primarily regulated by Ca2+–calmodulin binding, although it can also respond to WSS activation through phosphorylation by protein kinase B [137]. Acute or prolonged high laminar shear stress leads to the increased transcription and activation of eNOS, thereby promoting vasodilation to prevent initial vascular injury. However, under pathologically low WSS conditions, ECs fail to express eNOS [138]. Khurana et al. demonstrated, using gene chip technology, that the intron-4 27-base-pair variable number tandem repeat polymorphism (eNOS 27 VNTR) in the eNOS gene can predict the susceptibility to rupture of IAs [139]. In another study, Khurana et al. used gene microarray technology for detection, and the data results indicate that the eNOS T-786C genotype may be a factor influencing the size of aneurysm rupture in populations with known IA [140]. Therefore, NOS may be an important source of reactive nitrogen species (RNS) and ROS in IA, potentially promoting aneurysm formation and progression through various mechanisms [141].

### 7.3. Extracellular Matrix-Associated Proteins

The extracellular matrix (ECM) is a complex three-dimensional network composed of fibers, gels, and minerals, constituting the fundamental supportive structure for cell survival within biological tissues [142]. Under normal physiological circumstances, cells constantly modify the ECM through processes such as rearrangement, degradation, chemical modification, and synthesis [143]. However, in pathological conditions, the ECM undergoes notable remodeling in reaction to various stimuli, involving the removal of structural and functional proteins, playing a critical role in the formation of IA (Table 2).

Collagen is the main component of the ECM, playing a crucial role in offering structural support and maintaining tissue morphology and mechanical characteristics. It regulates cell growth, activity, and specialized development [144]. Collagen degradation is primarily regulated by MMPs. MMPs are accountable for the degradation of collagen types I, II, and III. Proteases including MMP-1 (interstitial collagenase), MMP-13 (collagenase-3), MMP-8 (neutrophil collagenase), and membrane-bound MMP-14 have the capability to degrade these collagen types. Specifically, MMP-2 is primarily responsible for degrading type I collagen; in contrast, MMP-13 is the enzyme of choice for degrading type II collagen, and MMP-8 and MMP-1 are mainly involved in the degradation of types I and III collagen [145]. The decrease in collagen content in the arterial wall is one of the prominent pathological features of IA [146]. Aoki et al. found that there was a decrease in collagen protein biosynthesis in the arterial aneurysm wall, representing an important aspect of the degenerative changes in the aneurysm wall. At the transcriptional level, the expression of type I and III procollagen in the aneurysm wall was downregulated [98]. In another study, they demonstrated that excessive collagen degradation in the wall of intracranial aneurysms is caused by an imbalance between MMPs and TIMPs as well as tissue proteases and cysteine protease C [147].

Elastin originates from tropoelastin, a soluble monomer crucial for maintaining the morphology, elasticity, and functionality of the ECM. It plays a critical supportive role physiologically [148]. Watton et al.’s aneurysm model suggests that aneurysm formation stems from the local degradation of elastin, leading to disturbances in arterial geometry. Further degradation of elastin is explicitly associated with low wall shear stress in specific regions of the artery, thereby promoting aneurysm development [149]. Nakagawa et al.’s analysis suggests that compared with unruptured aneurysms, the plasma concentration of soluble elastin fragments in the lumen of ruptured aneurysms is significantly higher [150]. A large-scale retrospective study in China reported that two single-nucleotide polymorphisms in the elastin gene are associated with the development and rupture of IAs, further emphasizing the role of elastin in aneurysm rupture [151]. Onda et al. found a strong association between variants in the elastin gene’s 20th intron/23rd intron (located on chromosome 7q11) and IA in Japan [152]. The measurement of elastin concentration in peripheral blood may be used to predict the risk of rupture in unruptured intracranial aneurysms.

Matrix metalloproteinases (MMPs) are a class of proteinases involved in the regulation of ECM by degrading proteins, regulating cell adhesion, and modulating cytokines [153]. MMPs were first discovered in 1962 and were initially identified as collagenases mediating the absorption of tadpole tails. This protein family shares structural similarities, and its enzymatic activity facilitates a range of biological processes. [154]. Most proteins found in the ECM are substrates of MMPs. MMPs and their degradation products have the ability to alter the downstream effects of cellular signaling molecules such as growth factors, cell adhesion molecules, cytokines, and other MMPs [155]. The regulation of MMP expression involves multiple factors, including inflammatory cytokines like TNF-α and IL-1β, vascular factors such as endothelin A, and ECM proteins [156]. TIMPs are the primary endogenous inhibitors of MMP activity, exerting their effects through binding to the C-terminal domain of MMPs [157]. In the development and pathogenesis of IA, MMPs and TIMPs play critical roles. MMPs belong to the protease family involved in vascular remodeling. Elevated activity of MMP-2, MMP-3, and MMP-9 within the arterial wall contributes to inflammation and remodeling of the extracellular matrix during IA [158]. Kim et al. used GWAS data to investigate the association between MMPs and IA. Three SNPs of MMP-24 were discovered to be significantly linked to IA, exerting a protective influence on IA development. They speculated that MMP-24 could impede IA formation by influencing ECM stiffness. Regarding MMP-13, they found a close association with IA, but it is unclear whether it facilitates or impedes IA formation. Additionally, they discovered MMP-2 to be the MMP most strongly associated with IA, with high expressions in vasculature, brain, and blood [159]. Aoki et al. reported that the elevated levels of MMP-2 and MMP-9, derived from macrophages within the IA wall, are closely associated with the progression of aneurysms [160]. Liu et al. found that in experimental cerebral aneurysms, the elastic fibers of the extracellular matrix were disrupted, leading to a reduction or loss of elastic fibers accompanied by extensive infiltration of inflammatory cells. The expression of MMP-9 in the aneurysm wall was significantly enhanced, and positive expression of MMP-9 was observed throughout the aneurysm wall. These results suggest that damage to elastic fibers is one of the key factors in aneurysm formation, while increased infiltration of inflammatory cells and secretion of MMP-9 are the main causes of elastic fiber damage [86]. The mechanism responsible for the increase in MMP levels during the development and rupture of IA remains uncertain. Zheng et al. found that compared with the control group, the mRNA expression levels of TNF-α, MMP-2, MMP-3, and MMP-9 were notably higher in IA patients. By delving deeper into the regulatory function of miR-513b-5p in the RIP1/RIP3/MLKL and MMP pathways in IA, they found that miR-513b-5p enhanced TNF-α-induced expression of MMP-2 and MMP-9 proteins. Additionally, they also discovered that the expression of TNF-α and MMP-9 was markedly elevated in patients with ruptured IAs in comparison with those with unruptured IAs. They concluded that TNF-α and MMP-9 have a greater impact on the rupture of IAs [56]. Yamaguchi et al. proposed that estrogen deficiency leads to decreased expression of ERα and Sirt1, resulting in reduced levels of the Sirt1 protein, which promotes the activation of NLRP3, thereby inducing the accumulation of IL-1β and MMP-9, potentially leading to the rupture of IA. Their research findings revealed an increase in IL-1β and MMP-9 protein levels in the rat IA rupture model, supporting the notion that IA rupture is at least partially associated with the activation of the NOD-, LRR-, and pyrin-domain-containing protein 3 (NLRP3) after ERα depletion in the rat IA model [93]. Yuan et al. found that CXC chemokine receptor 3 (CXCR3) is a direct target of miR-34a, which modulates the phenotypic transformation of VSMCs in IAs by directly targeting CXCR3. CXCR3 modulates the expression of MMP-2 through ceRNA regulation by sharing overlapping miR-34a binding sites [72].

## 8. Conclusions

The formation, development, and rupture of IAs are complex processes influenced by both genetic and environmental factors. Despite significant research, the underlying mechanisms remain incompletely understood. However, the exploration of genetic and molecular markers associated with IA offers new avenues for identifying high-risk populations. Numerous studies have shown a strong correlation of the levels of inflammatory markers like MCP-1, IL-1β, and TNF-α with different stages of IA development. Similarly, oxidative stress proteins such as COX-2, NOX, and NOS are believed to play critical regulatory roles in IA pathogenesis.

Moving forward, large-scale, multicenter studies are needed to validate the sensitivity and specificity of these biomarkers, particularly across diverse racial populations, as previous studies may have been limited by sample size. Additionally, the development of non-invasive and cost-effective screening tools holds promise for predicting IA formation, growth, and rupture risk, ultimately facilitating early intervention and improved patient outcomes.

## Data Availability

Data are contained within the article.

## Abbreviations

AA	Abdominal aortic aneurysm
BCL2	B-cell lymphoma-2
CASP1/ICE	Caspase 1
CD14	Cluster of differentiation 14
CNS	Central nervous system
COL1A1	Collagen type I alpha 1 chain
COL1A2	Collagen type I alpha 2 chain
COL3A1	Collagen type III alpha 1 chain
COL5A1	Collagen type V alpha 1 chain
COL5A2	Collagen type V alpha 2 chain
COX-1	Cyclooxygenase-1
COX-2	Cyclooxygenase-2
CSF	Cerebrospinal fluid
CTA	Computed tomography angiography
CXCL12	Chemokine (C-X-C motif) ligand 12
CXCR3	CXC chemokine receptor 3
DNMT3A	DNA methyltransferase 3 alpha
DPP-4	Dipeptidyl peptidase-4
DSA	Digital subtraction angiography
ECM	Extracellular matrix
ECs	Endothelial cells
EDNRA	Endothelin receptor type A
ELISA	Enzyme-linked immunosorbent assay
eNOS	Endothelial nitric oxide synthase
ET-1	Endothelin-1
EVs	Extracellular vesicles
FAS	Fatty acid synthase
FTO	Fat mass and obesity-associated protein
GEO	Gene expression omnibus
gp130	Glycoprotein 130
GREM1	Gremlin 1
GSTA4	Glutathione S-transferase alpha 4
GWAS	Genome-wide association study
H_2_O_2_	Hydrogen peroxide
HBVSMCs	Human brain vascular smooth muscle cells
HDAC9	Histone deacetylase 9
IA	Intracranial aneurysm
IAs	Intracranial aneurysms
IEL	Internal elastic lamina
IFN-γ	Interferon-gamma
IGF2BP1	Insulin-like growth factor 2 mRNA binding protein 1
IGF2BP3	Insulin-like growth factor 2 mRNA binding protein 3
IL-1β	Interleukin-1β
IL-6	Interleukin-6
IL-8	Interleukin-8
iNOS	Inducible NOS
KLF5	Kruppel-like factor 5
LOX	Lipoxygenase
KDM1A	Lysine-specific demethylase 1
LRPPRC	Leucine-rich pentatricopeptide repeat containing
m1A	N1-methyladenosine
m5C	2′-O-methylation, 5-methylcytosine
m6A	N6-methyladenosine
MAP3K10	Mitogen-activated protein kinase 10
MBD2	Methyl-CpG-binding domain protein 2
MCL-1	Myeloid leukemia 1
MCP-1	Monocyte chemoattractant protein-1
Methyl	Methylation
MIRA-seq	Methylated-pyrosequencing
MMP-2	Matrix metalloproteinase-2
MMP-3	Matrix metalloproteinase-3
MMP-9	Matrix metalloproteinase-9
MMP-13	Matrix metalloproteinase-13
MMPs	Matrix metalloproteinases
MRA	Magnetic resonance angiography
NADPH	Nicotinamide Adenine Dinucleotide Phosphate
NFATC1	Nuclear factor of activated T cell
NF-κB	Nuclear factor kappa-B
NLRP3	The NOD-, LRR-, and pyrin domain-containing protein 3
nNOS	Neuronal NOS
NO	Nitric oxide
NOS	Nitric oxide synthase
NOX	Nicotinamide Adenine Dinucleotide Phosphate Oxidase
O_2_^•-^	Superoxide
ONOO^•-^	Peroxynitrite
PCA	Principal component analyzed
PGE2	Prostaglandin E2
PGG2	Prostaglandin G2
PMN	Polymorphonuclear neutrophils
PNPLA6	Patatin-like phospholipase domain-containing protein 6
PTEN	Phosphatase and tensin homolog
PTGS2/COX2	Cyclooxygenase-2
qRT-PCR	Quantitative real-time polymerase chain reaction
RBM15	RNA-binding motif protein 15
RIA	Ruptured intracranial aneurysm
RNA-seq	RNA sequencing
RNS	Reactive nitrogen species
ROS	Reactive oxygen species
SAH	Subarachnoid hemorrhage
SERPINA3	Serine protease inhibitor clade A member 3
SMC	Smooth muscle cell
SMCs	Smooth muscle cells
SNPs	Single nucleotide polymorphisms
SOCS3	Cytokine signaling suppressor 3
TAM	Tumor-associated macrophage
TIMP4	Tissue inhibitor of metalloproteinase 4
TNC	Tenascin C
TNF-α	Tumor necrosis factor-alpha
TRPC6	Transient receptor potential-6
UIA	Unruptured intracranial aneurysm
VCAM-1	Vascular Cell Adhesion Molecule 1
VSMC	Vascular smooth muscle cell
WB	Western blotting
WSS	Wall shear stress
YTHDC1	YT521-B homology domain-containing protein 1
YTHDF2	YT521-B homology domain family member 2
YTHDF3	YT521-B homology domain family member 2
ZNF217	Zinc finger protein 217
